# A Novel 3D Culture Scaffold to Shorten Development Time for Multicellular Tumor Spheroids

**DOI:** 10.3390/ijms232213962

**Published:** 2022-11-12

**Authors:** Cian-Ru Yang, Chu-Ting Liang, Shih-Chieh Tsai, Yu-Chun Wu, Ching-Wen Liu, Hui-Hua Yang, Ting-Yuan Tu, Yueh-Chun Lee, Kuei-Yang Hsiao, Wei-Chun Chang, Wen-Lung Ma

**Affiliations:** 1 Ph.D. Program for Health Science and Industry, China Medical University, Taichung 404, Taiwan; 2 Department of Medical Research, Department of Obstetrics and Gynecology, China Medical University Hospital, Taichung 404, Taiwan; 3 GEcoll Biomedical Co., Ltd., Tainan Science Park, Tainan 744, Taiwan; 4 Bioinnovation Center, Buddhist Tzu Chi Medical Foundation, Hualien 970, Taiwan; 5 Department of Medical Research, Buddhist Tzu Chi General Hospital, Hualien 970, Taiwan; 6 Department of Biomedical Engineering, National Cheng Kung University, Tainan 701, Taiwan; 7 School of Medicine, Chung Shan Medical University, Taichung 402, Taiwan; 8 Institute of Biochemistry, College of Life Sciences, National Chung Hsing University, Taichung 402, Taiwan; 9 Department of Nursing, Asia University, Taichung 413, Taiwan

**Keywords:** 3D scaffold culture, multicellular tumor spheroid, cancer stem cell

## Abstract

Multicellular tumor spheroids and tumoroids are considered ideal in vitro models that reflect the features of the tumor microenvironment. Biomimetic components resembling the extracellular matrix form scaffolds to provide structure to 3-dimensional (3D) culture systems, supporting the growth of both spheroids and tumoroids. Although Matrigel has long been used to support 3D culture systems, batch variations, component complexity, and the use of components derived from tumors are complicating factors. To address these issues, we developed the ACD 3D culture system to provide better control and consistency. We evaluated spheroid and tumoroid formation using the ACD 3D culture system, including the assessment of cell viability and cancer marker expression. Under ACD 3D culture conditions, spheroids derived from cancer cell lines exhibited cancer stem cell characteristics, including a sphere-forming size and the expression of stem cell marker genes. The ACD 3D culture system was also able to support patient-derived primary cells and organoid cell cultures, displaying adequate cell growth, appropriate morphology, and resistance to oxaliplatin treatment. These spheroids could also be used for drug screening purposes. In conclusion, the ACD 3D culture system represents an efficient tool for basic cancer research and therapeutic development.

## 1. Introduction

The recent development of 3-dimensional (3D) culture systems for mammalian cells has revealed various cell biology features [1]. Compared with conventional 2-dimensional (2D) culture methods, 3D culture is considered a better model for both basic and clinical studies, as most mammalian cells grow and function in 3D environments in vivo. Multicellular tumor spheroids (MCTSs) are cancer cell aggregates grown in suspension or embedded in gels using 3D culture methods [1]. Previous studies have used tumor-derived spheroids to study cancer stem cells (CSCs) in vitro [1,2,3,4]. Tumor-derived spheroids are enriched in cells with CSC characteristics [5], including the expression of CSC-specific marker genes *CD24*, *CD44*, *CD133*, *Oct-4*, *Sox-2,* and *Nanog* [6,7]. Enriched CSC populations in spheroids display drug resistance to conventional chemotherapies [2,8,9]. Spheroid cell cultures can be used to study tumor cell signaling, changes in the tumor microenvironment, and the basic properties of CSCs [3]. Primary cancer cells can also be cultured into tumoroids using 3D culture systems for both basic research and anti-cancer drug screening [10]. Combined with genomic testing and patient-derived xenograft animal models, tumoroids may represent a cost-effective and efficient model for improving the development and evaluation of future anti-cancer drugs [11,12].

Several 3D culture methods have been developed for the generation of MCTSs, including static suspension, hanging drop, rotating bioreactor, magnetic levitation, electrical force assistance, and matrix-based hydrogels [1,10,13,14]. Matrix-based hydrogels utilize various biological materials that form scaffolds, combining mechanical strength and biochemical properties that resemble those found in the extracellular matrix (ECM) to provide physical support and promote cell growth, facilitating the formation of a 3D spheroid structure [15,16].

Natural materials, such as collagen [17,18], hyaluronic acid [17,19], chitosan, and alginate [20,21,22] have been used as biomimetic scaffolds for 3D spheroids grown from cancer and stromal cells [1]. Matrigel is a widely used scaffold material for 3D cultures using many cell types. Matrigel is derived from the basement membrane of Engelbreth–Holm–Swarm mouse tumor cells, composed of collagen IV, laminin, entactin, and multiple cytokines and growth factors [23,24,25]. Previous studies have examined the CSC properties of 3D culture systems using Matrigel. However, Matrigel is expensive, and its complex composition has led to manufacturing inconsistencies that have been linked to inconsistencies in study outcomes [25,26,27]. In addition, freeze–thaw cycles can lead to inconsistencies in the aqueous-to-solid phase of Matrigel during resolidification processes [27]. These disadvantages make the use of Matrigel for spheroid cell culture experiments challenging.

In this study, we developed a scaffold system using alginate, a natural polysaccharide extracted from brown algae [21,28] that is widely used in medical implants and various cell culture scaffolds [21,28]. A 3D scaffold model using alginate was previously used to study the Wnt/β-catenin signaling pathway in liver CSCs. The cells formed spheroids and maintained the functional structure and characteristics of CSCs [29]. However, alginate lacks the cell adhesion peptide Arg–Gly–Asp (RGD), which is essential for attachment between cells and the surrounding ECM [21,28]. Gelatin, a denatured collagen derived from connective tissue and contains the RGD sequence, is commonly added to alginate gels to interact with integrins on the cell membrane [30,31]. Cell attachment is the initial step in a cascade of cell–biomaterial interactions and is important for various cellular processes, including cell guidance, proliferation, and differentiation [31].

In the current study, we tested the efficacy and sphere-forming capabilities of the ACD 3D culture system using human carcinoma cell lines. In the ACD 3D culture system, gelatin is rapidly cleared, and the procedures are easy to follow.

## 2. Results

### 2.1. Preparation of the ACD 3D Culture System

**Figure 1:**
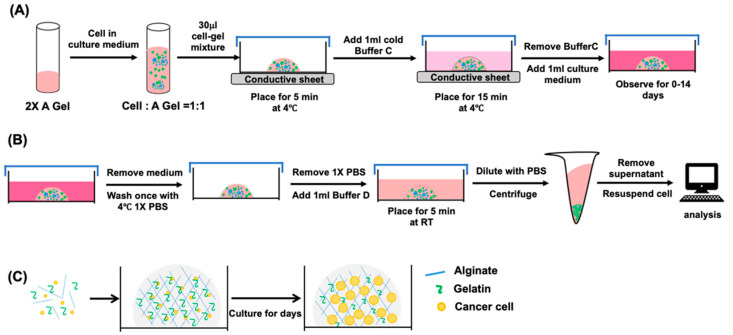
The procedures and principles underlying the ACD 3D culture system. (**A**) The process of gel formation. Cells suspended in culture medium are mixed with A gel, and 30 μL of the cell–gel mixture were plated on a 24-well plate. After 5 min of reaction on ice and 15 min of reaction with C buffer to promote gel cross-linking, the gel becomes stable and flexible and can maintain and support cell growth for up to 14 days. (**B**) The process for dissolving the gel to collect spheroid cells. The culture medium is removed, and the gel is washed once with 1× PBS. D buffer is added and incubated for 5 min to dissolve the gel. D buffer is diluted with 3 volumes of 1× PBS, followed by centrifugation for 10 min. Finally, cells are resuspended in 1× PBS for analysis. (**C**) The principle mechanism of the ACD 3D culture system. Alginate and gelatin are the main components of A gel. Alginate forms the scaffold, and gelatin provides a cell adhesion motif for cancer cells and is released into the culture medium over time.

Cells were cultured in the ACD 3D cultures system as described in Materials and Methods and illustrated in Figure 1A,B. Gel formation occurred after 20 min, which is faster than the 30 min required for Matrigel. In the ACD system, A gel contains cross-linked alginate that forms a scaffold structure and gelatin to provide the initial cell adhesion sequences. The gelatin is gradually released into the culture medium, providing more space for spheroid cells to grow (Figure 1C).

### 2.2. Basal Cell Growth Profile of Cell Lines in 2D Culture

To evaluate the cell growth rate for cells grown in the ACD 3D culture system, the basal cell growth rate (calculated as the doubling time) was determined for cells grown in 2D culture. Various cancer cell lines, including AGS (gastric cancer (GCa)), SKOV3, MDAH-2774 (epithelial ovarian cancer (EOC)), PanC-1 (pancreatic ductal adenocarcinoma (PDAC)), and HepG2 (hepatocellular carcinoma (HCC)) cells were examined (Table 1). The doubling times for AGS and MDAH-2774 cells were 15.9 h and 24.6 h, respectively, which were both faster than the doubling times for PanC-1 (29.4 h), HepG2 (37.7 h), and SKOV3 (39.4 h) cells. A shorter doubling time indicates faster cell proliferation, providing basal information for evaluating the rate of spheroid formation in the ACD 3D culture system.

### 2.3. Fast and Viable Spheroid-Forming Performance in ACD 3D-Culture System

**Figure 2:**
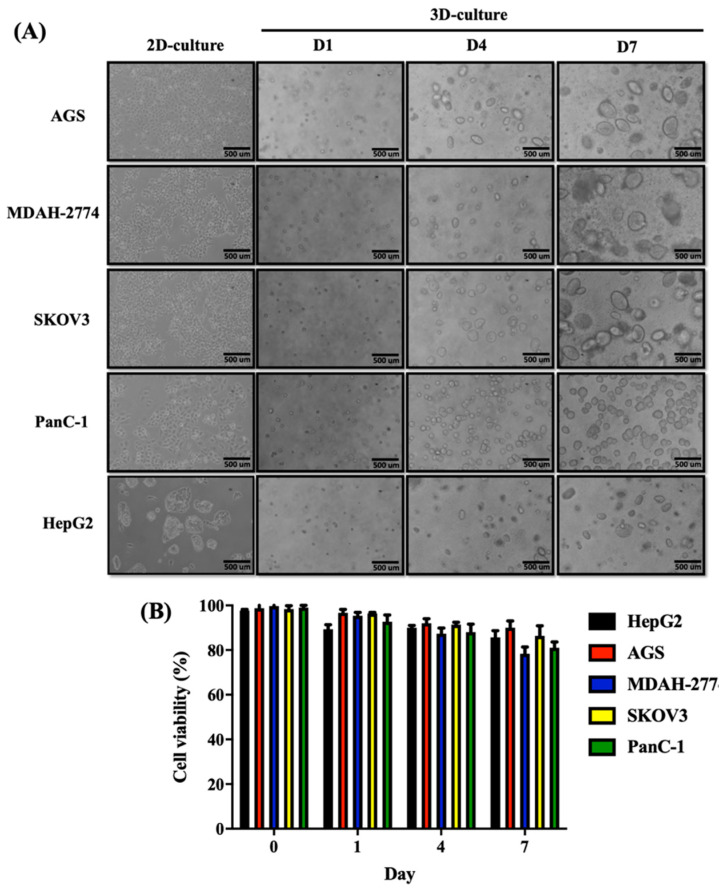
Spheroid formation and cell viability of cancer cell lines cultured using the ACD 3D culture system. (**A**) Five different cancer cell lines, AGS, MDAH-2774, SKOV3, PanC-1, and HepG2 cells were cultured for 3 days in 2D culture. Bright-field images show the morphologies of cells in adherent culture. AGS, MDAH-2774, SKOV3, PanC-1, and HepG2 cells can form spheroids when 3000 cells are seeded into 24-well plates within 4 days after incubation in the ACD 3D culture system. Bright-field images show the morphologies of spheroids on Days 1, 4, and 7 in the ACD 3D culture system. On Day 7, AGS, MDAH-2774, and SKOV3 cells formed large, ellipsoid spheroids, whereas HepG2 cells formed small, ellipsoid spheroids and PanC-1 cells formed small, round spheroids. Images were captured using phase-contrast microscopy on Days 1, 4, and 7. Scale bar = 500 µm. (**B**) Spheroids were collected on Days 1, 4, and 7. After resuspension of spheroids to obtain single-cell suspensions, cell viability was evaluated by 0.2% trypan blue. Cells obtained from AGS, MDAH-2774, PanC-1, and HepG2 spheroids exhibited greater than 80% viability on Days 1 and 4. On Day 7, the cell viabilities of AGS, MDAH-2774, PanC-1, and HepG2 cells were greater than 70%. Data shown as mean ± SD of triplicate independent experiments.

To evaluate the spheroid-forming capacity and efficiency of the ACD 3D culture system, we evaluated the sphere-forming times and morphologies of 5 cancer cell lines, including AGS (GCa), SKOV3, MDAH-2774 (EOC), PanC-1 (PDAC), HepG2 (HCC) cells. Regardless of the basal cell growth rate, all 5 human carcinoma cell lines examined were able to form spheroids. We observed that fast-growing cancer cell lines were able to form spheroids rapidly when grown using the ACD 3D culture system. The fastest growing cell lines, AGS and MDAH-2774 cells, formed spheroids rapidly by Day 3, whereas PanC-1, HepG2, and SKOV3 cells formed spheroids by Day 4 (Figure 2A, Table 2). Spheroid morphologies and sizes differed according to cancer type. AGS, SKOV3, and HepG2 cells formed spheroids that were ovoid, whereas PanC-1 and MDAH-2774 spheroids were round. We also observed variations in spheroid size, with AGS, MDAH-2774, and SKOV3 cells forming larger spheroids than those formed by HepG2 and PanC-1 cells (Figure 2A).

Next, we evaluated cell viability during both early and late stage spheroid formation. Cell viability was approximately 90% during early spheroid formation. At Day 7 of culture, the cell viabilities were greater than 70% for AGS (90%), MDAH-2774 (75%), PanC-1 (84%), HepG2 (90%), and SKOV3 (91%) cells (Figure 2B, Table 2). These results indicate that the ACD 3D culture system can support long-term spheroid growth. Taken together, these data indicate that the ACD 3D culture system not only provides suitable conditions for rapid spheroid formation and growth but also for maintaining long-term cell viability.

### 2.4. Cancer Stem Cell Marker Expression in Spheroids Grown Using the ACD 3D Culture System

**Figure 3:**
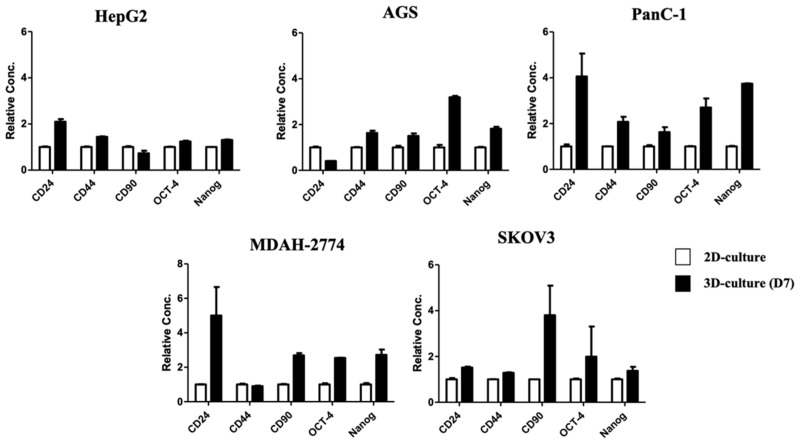
Spheroids derived from cancer cell lines cultured using the ACD 3D culture system exhibit cancer stem cell characteristics. Cells grown in 2D culture cells and spheroids grown in 3D culture were collected on Day 7 for mRNA extraction. mRNA levels of the stemness markers *CD24*, *CD44*, *CD90*, *Oct-4*, and *Nanog* were examined in parent and spheroid cells using Real-time PCR. The white bars show the levels measured in cells from 2D culture, and the black bars show the level measured in spheroids grown in 3D culture. All gene expression levels were normalized against the expression level of β-actin. All results are expressed as the fold change relative to the expression level in cells grown in 2D culture. Data shown as mean ± SD of triplicate independent experiments.

Previous studies described spheroids as displaying CSC characteristics. We evaluated whether spheroids cultured using the ACD 3D culture system expressed CSC markers. After 7 days of culture, we collected spheroids and extracted RNA to examine the expression of *Oct-4*, *Nanog*, *CD24*, *CD44,* and *CD90* using Real-time PCR. CSC marker gene expression varied across different cancer cell types. In HepG2 cells, *CD24* expression in spheroids differed by 2-fold compared with cells grown in 2D culture. In AGS cells, *CD44*, *CD90*, *Oct-4*, and *Nanog* expression levels increased in spheroids compared with cells grown in 2D culture. PanC-1 and SKOV3 cells expressed *CD24*, *CD44*, *CD90*, *Oct-4*, and *Nanog* when grown in ACD 3D cultures. MDAH-2774 cells express higher levels of *CD24*, *CD90*, *Oct-4*, and *Nanog* when grown in spheroids than when grown in 2D cultures (Figure 3). Taken together, these results indicate that cancer cells cultured using the ACD 3D culture system are able to form spheroids that express CSC-related genes, indicating that cells grown using the ACD 3D culture system may be useful for CSC research.

### 2.5. Anti-Cancer Drug Effects on Spheroids Grown Using the ACD 3D Culture System

**Figure 4:**
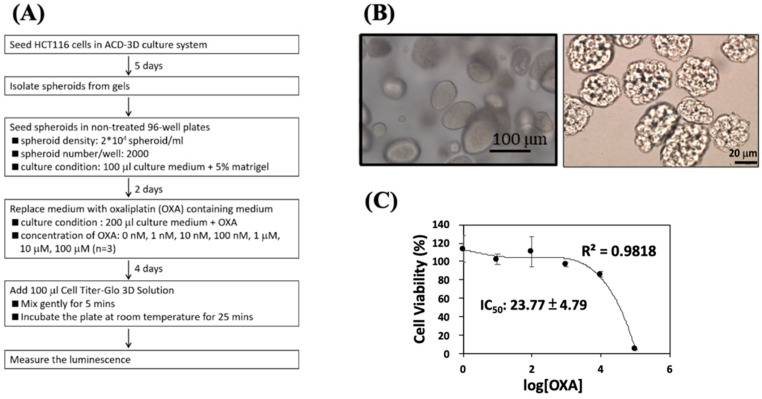
The anti-cancer drug test was performed in HCT116 spheroids grown using the ACD 3D culture system. (**A**) A flowchart showing the anti-cancer drug test procedures performed using cells cultured in the ACD 3D culture system. (**B**) HCT116 spheroid formation using the ACD 3D culture system (**left**, 20×) and the spheroids collected from gels (**right**, 40×). (**C**) After 4 days of OXA treatment, spheroid cell viability was examined using Cell Titer-Glo^®^ 3D Cell Viability Assay. The IC_50_ of oxaliplatin (OXA) is 23.77 ± 4.97 μM in HCT116 spheroids grown using the ACD 3D culture system. Data shown as mean ± SD of triplicate independent experiments.

Spheroids and tumoroids grown in 3D culture have previously been used as anti-cancer drug screening models [10]. To test whether spheroids grown using the ACD 3D culture system could be utilized for anti-cancer drug screening purposes, HCT116 colon cancer cells grown using the ACD 3D culture system were harvested to examine their response to the anti-cancer drug OXA (Figure 4A). HCT116 spheroids grow well in the ACD 3D culture system for 5 days (Figure 4B). ACD gels containing embedded HCT116 spheroids were dissolved with D buffer, and spheroids were collected. Spheroids were cultured in untreated wells for 2 days in medium containing 5% Matrigel to maintain the spheroid structure. After 4 days of OXA treatment, the cell viability was examined. The half-maximal inhibitory concentration (IC_50_) for OXA is 23.77 ± 4.97 μM in HCT116 spheroids (Figure 4C). The ACD 3D culture system can be used as a tool for drug screening.

### 2.6. Primary Cells from Patient-Derived Cancer Tissue Grown Using the ACD 3D Culture System

**Figure 5:**
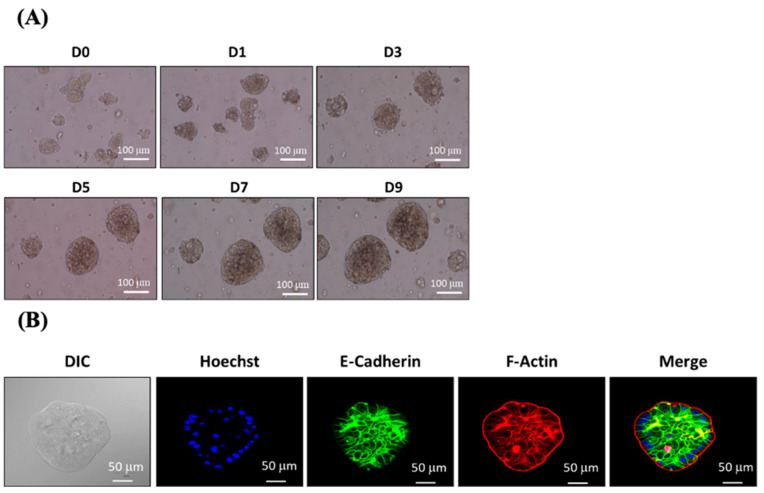
Primary colon cancer cells from patient-derived tissue samples were grown using the ACD 3D culture system. (**A**) Primary colon cancer cells obtained from patient-derived tissue samples were trypsinized and cultured using the ACD 3D culture system for 9 days. Bright-field images show the morphologies of spheroids grown in the ACD 3D culture system on Days 0, 1, 3, 5, 7, and 9. Images were captured using a phase-contrast microscope. Scale bar = 100 µm. (**B**) The expression of cancer markers E-Cadherin and F-actin in primary colon cancer tumoroids. Tumoroids isolated from ACD 3D culture system gels were subjected to immunofluorescent staining. Images were captured using a laser scanning confocal microscope at the magnification of 400×. Blue: Hoechst 33258; green: E-Cadherin; red: F-actin. Data shown from triplicate independent experiments.

To examine whether the ACD 3D culture system can support the formation of tumoroids, primary cells isolated from patient-derived colon cancer samples were seeded on ACD gels. This study was approved by the Institutional Review Boards at the Chung Shan Medical University Hospital (CS1-20040). When cultured using an appropriate culture medium, patient-derived colon cancer cells steadily grew and formed tumoroids starting on Day 3 of the 3D culture (Figure 5A). Cells in the center of the mass became dark starting on Day 10 most likely due to insufficient nutritional penetration from the culture medium. To further confirm that the observed masses were derived from colon cancer cells, the gel was dissolved with D buffer, and the masses were isolated intact. The expression of colon cancer markers, including E-Cadherin and F-actin, were examined in the isolated cell masses (Figure 5B). E-Cadherin is localized at cell–cell junctions, whereas F-actin is distributed not only at cell–cell junctions but also at the edges of the cell mass. The expression and distribution of E-Cadherin and F-actin indicate that the cell masses represent tumoroids derived from primary colon cancer cells.

### 2.7. Human Liver Organoid Formation with ACD 3D-Culture System

**Figure 6:**
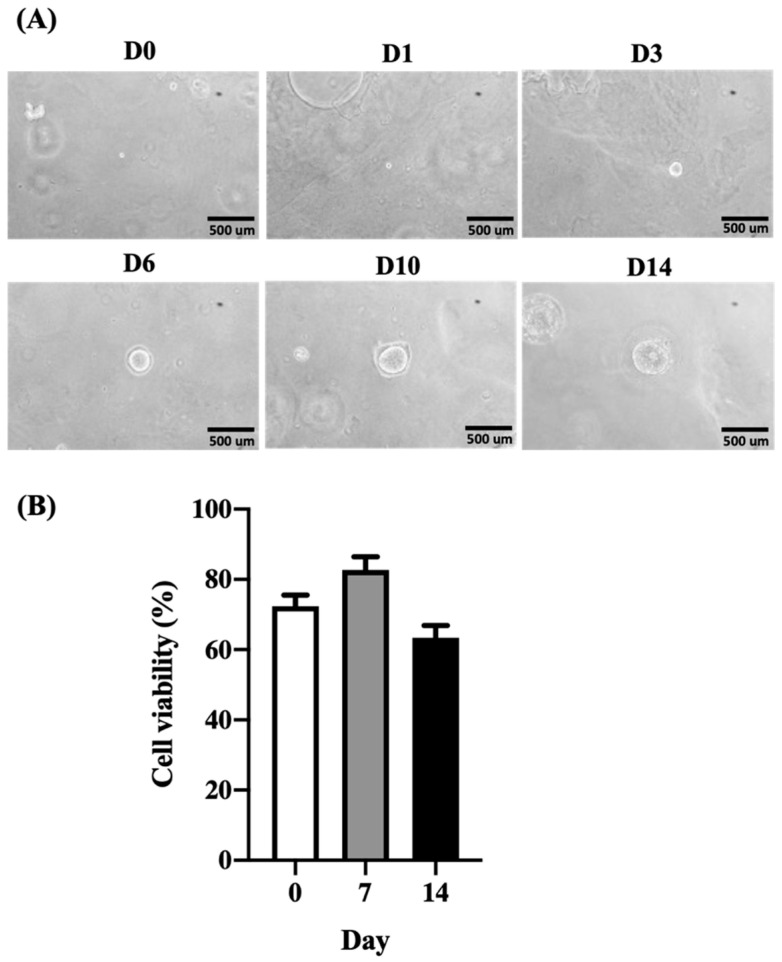
Human liver organoid formation and cell viability when cultured using the ACD 3D culture system. (**A**) Thawed single-cell organoid suspensions are able to regenerate organoids when grown using the ACD 3D culture system, starting at Day 6, and growing stably until Day 14. Bright-field images show the morphologies of organoids on Days 0, 1, 3, 6, 10, and 14. Organoids appeared round. Images were captured using a phase-contrast microscope. Scale bar = 500 µm. (**B**) Organoids were collected on Days 0, 7, and 14 and resuspended into single-cell suspensions. Cell viability was evaluated by 0.2% trypan blue. Cell viability was greater than 80% on Day 7. On Day 14, cell viability was greater than 50%. Data shown as mean ± SD of triplicate independent experiments.

Organoid culture is an in vitro system intended to recapitulate tissues in a culture dish. Organoids derived from healthy donors or patients can be used as research models to investigate healthy liver development and disease mechanisms [32]. Liver organoids can also be used to perform drug screening and assess gene therapy [32]. To evaluate whether the ACD 3D culture system can support organoid growth, we thawed previously stored, organoid-derived, single-cell suspensions for culture using the ACD 3D culture system. Organoid-derived, single-cell suspensions were able to regenerate into organoids when cultured using the ACD 3D culture system, with organoids appearing on Day 6 and stable growth observed until Day 14 (Figure 6A). Next, we evaluated the cell viability of organoid cells. Single-cell viability was 76% when measured using freshly thawed cells. After culture using the ACD 3D culture system, cell viability increased to 86% by Day 7, and 50% cell viability was maintained at Day 14. Therefore, the ACD 3D culture system can support organoid cell growth and survival.

## 3. Discussion

Several 3D culturing methods have been developed, including suspension culture, hanging drop culture, and matrix-based gels. Suspension culture uses a serum-free medium containing higher concentrations of growth factors [1,3], in which cells are grown in low-adhesion conditions, preventing migration. However, spheroids grown in suspension culture do not provide a good representation of the tumor microenvironment, and cell viability is low for long-term suspension cultures, making them difficult to passage [33]. The hanging drop method is another type of suspension culture. Briefly, a small cell volume is placed onto a culture plate, and the plate is inverted to create droplets. Cells will aggregate and form a spheroid at the drop tip [1,34]. Although this method is simple, it also has disadvantages. The droplet must be prevented from falling, and once the droplet begins to form, the removal or replacement of the medium becomes difficult. These disadvantages limit the applications of hanging drop cultures. Hydrogels are widely used in 3D cultures because they provide a scaffold structure and ECM-like properties to promote cell adhesion during the spheroid initiation stage [16].

In general, scaffold materials can be categorized into two types: natural polymers, such as collagen, chitosan, hyaluronic acid, fibroin, agarose, and alginate; and synthetic polymers, such as polyglycolic acid, polylactic acid, and aliphatic polyester polycaprolactone. Natural polymers have reduced toxicity and can improve biocompatibility compared with synthetic polymers. Although synthetic polymers have higher reproducibility and easier production procedures, they lack bioactivity [10].

The ACD 3D culture system utilizes a combination of alginate and gelatin to provide a scaffold structure. Alginate and gelatin are both natural materials that are easily obtained at a low cost. Alginate is commonly used in hydrogels to provide a scaffold structure. However, alginate lacks a cell adhesion motif [30], and cells do not express receptors that recognize alginates [28]. Therefore, we used gelatin to provide a cell adhesion signal. In addition, due to the physical properties of gelatin, gelatin is slowly secreted from the hydrogel into the culture medium over time.

Achieving good conditions for spheroid formation and growth requires the optimization of scaffold porosity, strength, structural stability, and degradation kinetics. The stiffness of hydrogels can affect the phenotypes and growth patterns of cancer cells. The numbers and sizes of cancer cell spheroids tend to be smaller when cultured in stiff hydrogels. We also observed the effects of hydrogel stiffness on spheroid formation. When using the ACD 3D culture system, proper stiffness helped to stablize spheroid cell formation than harder gels [16,35]. Therefore, the stiffness of hydrogels for optimization by modifying the concentrations of the composition materials was conducted.

Alginates are linear copolymers composed of two building units, β-D-mannuronic (M) acid and α-L-guluronic (G) acid, and the entire molecule consists of MM or GG monomeric or MG dimeric blocks [36]. High viscosity is achieved through increased M concentrations, whereas increased G concentrations result in stronger gelling properties. Alginate has a high affinity for alkaline earth metals, and hydrogels can form in the presence of divalent cations. The affinity of alkaline earth metals upon alginate gel is varied [36,37,38]. In the ACD 3D culture system, we use alkaline metals to achieve alginate gel cross-linking, resulting in a gel with low viscosity and high flexibility. The flexible alginate gel was combined with gelatin to promote stable cell adhesion on the scaffold, allowing for the rapid formation of spheroids.

Spheroids were enriched in cells with CSC characteristics [5]. In a previous study, multiple CSCs were successfully enriched by culturing them with alginate gel beads [22], suggesting that alginate gel may represent a useful biomaterial for enriching CSCs in culture. Our ACD 3D culture system results demonstrated similar findings. In the spheroids experiment using the ACD 3D culture system, we’ve examined the expression of CSC marker genes, such as CD24, CD44, CD90, and stemness-related transcriptional factor Nanog and Oct-4. It’s known that Nanog and OCT4 expressed in many cancer types [39]. High expression of Oct-4 has been associated with patients of poor prognosis as well [39]. High expression of Nanog promote the epithelial-mesenchymal transition (EMT) and improve cancer progress development. CD24, CD44 and CD90 are stemness-related surface marker and the most widely used markers in CSC research. [40,41].

High expressed Oct-4 and Nanog in Spheroids of 5 cancer types with ACD system indicating CSC characteristics. In the HCC, CD24 and CD44 are related to drug resistance and tumorigenesis. CD44 is also a cell surface marker for identification of gastric CSC. In ACD system, spheroids of AGS express CD44, which is consistent with previous research [39,42]. In pancreatic cancer, CD24 and CD44 are recognized as malignant CSC marker [39,43,44]. The PanC-1 spheroid exhibited high level of CD24 and CD44, indicating CSC was enriched in the culture. CD90 has been reported as a marker of ovarian CSCs and highly expressed in epithelial ovarian cancer [39,45]. Our data showed that SKOV3 and MDAH-2774 exhibited high CD90 expressed in spheroid, which is consistent with previous research. In sum, using 5 cancer types with ACD system without the stimulation of additional growth factors, the expression of CSC markers demonstrated the calibers ACD system for in vitro stemness study.

Spheroids display higher drug resistance due to the exclusion of drugs and the higher concentration of hypoxic cells that results from the gradient of nutrients and oxygen able to reach the outer and inner layers [46]. In this study, HCT116 spheroids were cultured using the ACD 3D culture system and tested for anti-drug efficacy. OXA is a chemotherapy drug commonly used to treat various cancers, including colon cancer. In the HCT116 drug-resistant colon cancer cell line, the OXA IC_50_ is typically 10–15 μM [47]. When HCT116 cells were cultured using the ACD 3D culture system, the OXA IC_50_ was 23.77 ± 4.97 μM. These data indicate that spheroids generated using the ACD 3D culture system may be useful tools for the future effective drug-screening.

The ACD 3D culture system not only supported spheroid formation from various cancer cell lines but also supported the generation of human cancer tumoroid and organoid cultures. In this study, primary cells isolated from patient-derived colon cancer samples successfully formed tumoroids when grown in the ACD 3D culture system. The ACD 3D culture system also supported human liver organoid growth, even when using freeze–thawed cells. The ACD 3D culture system supported the stable growth of organoid cells and maintained cell viability in long-term culture.

## 4. Materials and Methods

### 4.1. ACD 3D Culture System Procedure

The ACD 3D culture system (purchased from GEcoll Co., Ltd., Tainan, Taiwan) is constituted with A gel, C buffer and D buffer. The A gel was prepared with 2% alginate (Sigma, Singapore), 10% gelatin (GEcoll, Tainan, Taiwan) and 0.01 M HEPES (Sigma, Singapore) in distill water. The C buffer was prepared with 0.5% SrCl_2_ (Sigma, Singapore) in distilled water. The D buffer was prepared with 2% EDTA (Sigma, Singapore) in distilled water. All the prepared reagent was adjusted to pH 7.4 and sterilized with autoclave.

All steps were performed in a laminar flow hood, and each component was added using the volumes or ratios indicated. A thermally conductive sheet was placed on an ice pack to facilitate the rapid and even cooling of the culture plate. Cells were resuspended in 500 µL culture medium and mixed with 500 µL A gel at a 1:1 ratio. A 24-well plate was placed on the thermally conductive sheet, and a 30 µL cell—gel mixture was added to each well and maintained 4 °C for 5 min to ensure gel formation. After gel formation, 1 mL cold 1× C buffer was added to each well to cover the gel and incubated for 15 min. After 15 min, the C buffer was carefully removed, and 1 mL culture medium was added. Cells were incubated at 37 °C in a 5% CO_2_ incubator. Cell viability and spheroid cell morphology were observed and recorded at Day 0, 1, 4, 7.

### 4.2. Spheroid Collection and Cell Viability

The experimental procedure was modified from previously described procedures [48]. After spheroids were cultured in a 24-well plate, the culture medium was carefully removed, and the gel was washed the gel with cold 1× phosphate-buffered saline (PBS), followed by the addition of 1 mL 1× D buffer to each well and incubation for 5 min at room temperature. After incubation, the solution was gently mixed with repeated pipetting until the gel was completely dissolved. The solution was then transferred to a new 50-mL tube, and 3× volumes of PBS were added. The tube was centrifuged at 200× *g* for 10 min. The supernatant was discarded, and the cells were resuspended in 500 µL PBS and 0.2% trypan blue was added to evaluate cell viability and perform cell counting.

### 4.3. Real-Time PCR

The experimental procedure was modified from previously described procedures [49]. Spheroid cells were collected on Day 7 and washed once with 1× PBS, followed by centrifugation at 1500 rpm for 5 min. Total RNA was extracted from cells grown in both 2D-culture and as spheroids using Trizol reagent (Cat: T9424, Sigma-Aldrich, St. Louis, CA, USA), following the manufacturer’s protocol [50]. mRNA expression levels were validated in triplicate by Q- PCR. Briefly, reverse transcription from 5 μg RNA was carried out using PrimeScript™ RT reagent kit (Cat: RR037A, TaKaRa, Tokyo, Japan), according to the manufacturer’s protocol [51]. Real-time PCR was performed using KAPA SYBR FAST qPCR Master Mix (KAPA Biosystems, KM4100) with the specific primer shown in Table 3 on a Bio-Rad CFX96 Real-Time System (Bio-Rad, Hercules, CA, USA). Gene expression levels were normalized against the expression level measured for actin and using the ΔΔCt method to quantify the relative gene expression [52].

### 4.4. Immunofluorescence

Cells from patient-derived primary colon cancer samples were trypsinized and seeded in the ACD 3D culture system with culture medium. After 9 days, cultured tumoroids were isolated from the gel using D buffer, according to the manufacturer’s instructions. Isolated tumoroids were fixed with 100% methanol for 1 h and washed twice with 1× PBS at room temperature. Fixed tumoroids were blocked with 1 mg/mL BSA at room temperature for 1 h. Tumoroids were then stained with anti-E-Cadherin primary antibody (Cat#3195, Cell Signaling) and 1 μM Andy Fluor 594-conjugated phalloidin (Cat#C054, GeneCopoiea) for F-actin staining at room temperature for 2 h, followed by two washes with 1x PBS. After 1 h incubation with Alexa Fluor 488-conjugated secondary antibody (Cat#111-545-003, Jackson ImmunoResearch) the samples were washed twice with 1× PBS. Residual 1× PBS was removed, and the tumoroids were mounted with mounting buffer and covered with cover glass for observation under a laser scanning confocal microscope at the magnification of 400×.

### 4.5. Drug Screening

HCT116 cells were cultured using the ACD 3D culture system in Dulbecco’s modified Eagle medium (DMEM) supplemented with 10% fetal bovine serum (FBS) for 5 days. Spheroids began forming on Day 2. After 5 days of incubation, grown spheroids were isolated using buffer D according to the ACD 3D culture system instructions. The concentration of isolated spheroids was measured, and 2000 spheroids were seeded into untreated 96-well plates containing 100 μL of DMEM + 10% FBS and 5% Matrigel. After 2 days of culture, the culture medium was replaced with DMEM medium + 10% FBS containing different concentrations of the anti-cancer drug oxaliplatin (OXA). After 4 days of OXA incubation, 100 μL Cell Titer-Glo^®^ 3D Cell Viability Assay (Promega, Cat# G9681) was added to each well, mixed gently, and incubated for 25 min at room temperature. The luminescence of each sample was examined with GraphPrism 8, and cell viability was determined. The set of spheroids cultured in the medium without OXA was considered as Blank. The IC_50_ values were performed using GraphPad Prism (Intuitive Software for Science, San Diego, CA, USA) and 95% confidence intervals were obtained by nonlinear repression.

### 4.6. Statistics

Data are displayed as the mean ± standard deviation and were analyzed with Prism version 8.0 (GraphPad Software). An unpaired Student’s *t*-test was used to perform comparisons between two groups. A *p*-value < 0.05 was considered significant.

## 5. Conclusions

These data demonstrate that the ACD 3D culture system represents a low-cost, simple, and easy-to-operate culture system. Optimizing the composition of the alginate hydrogel could improve the efficiency and quality of spheroid research. In the future, the ACD 3D culture system can be more widely used when performing 3D culture studies.

## Figures and Tables

**Table 1:** The doubling time for different cancer types.

Cancer Type	Cell Line	Doubling Time (h)
Gastric cancer	AGS	15.9
Epithelial ovarian cancer, endometrioid type	MDAH-2774	24.7
Pancreatic ductal adenocarcinoma	PanC-1	29.4
Hepatocellular carcinoma	HepG2	37.7
Epithelial ovarian cancer, serous type	SKOV3	39.4

**Table 2:** Characteristics of different cancer cell lines grown in 3D culture.

Cancer Type	Cell Line	Doubling Time (h)	Time to Spheroid Formation (Days)	Cell Viability (%)
Gastric cancer	AGS	15.9	3	90
Epithelial ovarian cancer, endometrioid type	MDAH-2774	24.7	3	75
Pancreatic ductal adenocarcinoma	PanC-1	29.4	4	84
Hepatocellular carcinoma	HepG2	37.7	4	90
Epithelial ovarian cancer, serous type	SKOV3	39.4	4	91

**Table 3:** Real-time PCR Specific Primers.

Primer		Sequence (5′–3′)
*β-actin*	Forward	TCACCCACACTGTGCCCATCTACGA
	Reverse	CAGCGGAACCGCTCATTGCCAATGG
*CD24*	Forward	TTTACAACTGCCTCGACACACATAA
	Reverse	CCCATGTAGTTTTCTAAAGATGGAA
*CD44*	Forward	GACCTCTGCAAGGCTTTCAA
	Reverse	TCCGATGCTCAGAGCTTTCTC
*CD90*	Forward	CTAGTGGACCAGAGCCTTCG
	Reverse	TGGAGTGCACACGTGTAGGT
*Oct-4*	Forward	GGCCCGAAAGAGAAAGCGAACC
	Reverse	ACCCAGCAGCCTCAAAATCCTCTC
*Nanog*	Forward	GGGCCTGAAGAAAACTATCCATCC
	Reverse	TGCTATTCTTCGGCCAGTTGTTTT

## Data Availability

All data relevant to the study are included in the article.
